# Awake dynamics and brain-wide direct inputs of hypothalamic MCH and orexin networks

**DOI:** 10.1038/ncomms11395

**Published:** 2016-04-22

**Authors:** J. Antonio González, Panagiota Iordanidou, Molly Strom, Antoine Adamantidis, Denis Burdakov

**Affiliations:** 1 The Francis Crick Institute, Mill Hill Laboratory, London NW7 1AA, UK; 2 MRC NIMR, London NW7 1AA, UK; 3 Sainsbury Wellcome Centre, UCL, London W1T 4JG, UK; 4 Department of Neurology, University of Bern, Bern University Hospital, 3010 Bern, Switzerland; 5 Department of Developmental Neurobiology, King's College London, London WC2R 2LS, UK

## Abstract

The lateral hypothalamus (LH) controls energy balance. LH melanin-concentrating-hormone (MCH) and orexin/hypocretin (OH) neurons mediate energy accumulation and expenditure, respectively. MCH cells promote memory and appropriate stimulus-reward associations; their inactivation disrupts energy-optimal behaviour and causes weight loss. However, MCH cell dynamics during wakefulness are unknown, leaving it unclear if they differentially participate in brain activity during sensory processing. By fiberoptic recordings from molecularly defined populations of LH neurons in awake freely moving mice, we show that MCH neurons generate conditional population bursts. This MCH cell activity correlates with novelty exploration, is inhibited by stress and is inversely predicted by OH cell activity. Furthermore, we obtain brain-wide maps of monosynaptic inputs to MCH and OH cells, and demonstrate optogenetically that VGAT neurons in the amygdala and bed nucleus of stria terminalis inhibit MCH cells. These data reveal cell-type-specific LH dynamics during sensory integration, and identify direct neural controllers of MCH neurons.

## Abstract (web summary)

Hypothalamic neurons expressing melanin-concentrating-hormone (MCH) maintain body weight by orchestrating behaviour and metabolism, but little is known about their intrinsic regulation. Here, Gonzalez and colleagues reveal their behaviour-related dynamics during wakefulness, and map their brain-wide neural inputs.

## Graphical abstract

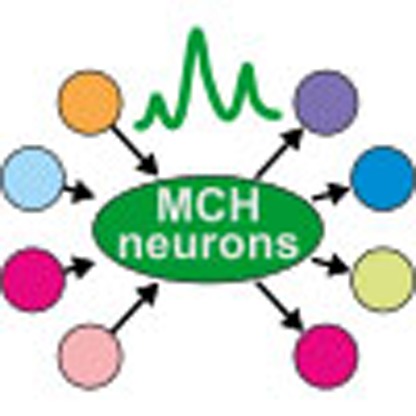

The lateral hypothalamus (LH) is critical for arousal and energy control in mammals; its lesions produce disorders of eating, body weight and sleep–wake regulation^1^,^2^. Decades ago, LH neurons were found to sense changes in internal^3^ and external^4^ environment, suggesting that the LH regulates physiology according to an integrated environmental state. However, owing to the functional diversity of LH neurons, it remains unclear how the LH sensory responses relate to neurochemically defined energy-related signals. Distinct LH projection neurons, such as the melanin-concentrating-hormone (MCH) and orexin/hypocretin (OH) cells, regulate distinct aspects of energy balance and vital behaviour^5^,^6^,^7^,^8^,^9^,^10^,^11^,^12^,^13^. Dysfunction of MCH neurons produces weight loss, indicating that they promote energy accumulation^5^,^6^,^13^. In contrast, deletion of OH neurons produces obesity, indicating that they promote energy expenditure^7^,^8^. MCH cells stimulate theta oscillations^9^ linked to memory formation^10^, participate in specific stages of sleep^9^ and are critical for learning to make choices that maximize body energy levels^11^. On the other hand, OH cells promote general arousal and ‘fight-or-flight' responses (reviewed in ref. 12). Both MCH and OH cells project widely to brain areas regulating attention, memory, reward, sleep/wake cycles and autonomic control^6^,^9^,^14^. However, the origins and functions of direct, monosynaptic neural inputs to MCH and OH neurons remain incompletely understood.

Previous work examining *in vivo* activity of MCH and OH cells provided fundamental insights into their physiological roles, yet key questions remain to be clarified. For example, recordings from a sample of MCH cells revealed cell firing during REM sleep but not wakefulness^15^. However, more recent studies found behavioural alterations upon experimental silencing of MCH cells in awake mice^11^, raising the question of whether some MCH cells are also active under certain conditions during wakefulness. In turn, recordings from OH cells^16^,^17^ showed rapid activation upon external disturbances, consistent with their proposed roles in ‘fight-or-flight' physiology^16^. However, the implications of this for MCH cell coordination are unclear, as existing *in vitro* data suggest that the OH cell activation can either excite^18^ or inhibit^19^ MCH cells.

The present study aims to address this missing information about LH neurons, by exploring (i) how MCH and OH population dynamics evolve in real-time during sensory experiences in awake mice; (ii) what brain centres directly innervate MCH and OH neurons and (iii) how genetically and anatomically defined input circuits shape MCH neuron activity. To achieve this, we perform deep-brain optical recordings of real-time MCH and OH cell activity in awake freely moving mice, and carry out retrograde and anterograde connectivity analyses of neural inputs to molecularly defined LH cells. We find differential modulation of MCH and OH cell activity during wakefulness, and show that MCH and OH cells receive direct neural inputs from multiple brain areas, including inhibitory inputs to MCH cells from the amygdala and bed nucleus of stria terminalis.

## Results

### Optical recordings from LH cells in freely moving mice

**Figure 1:**
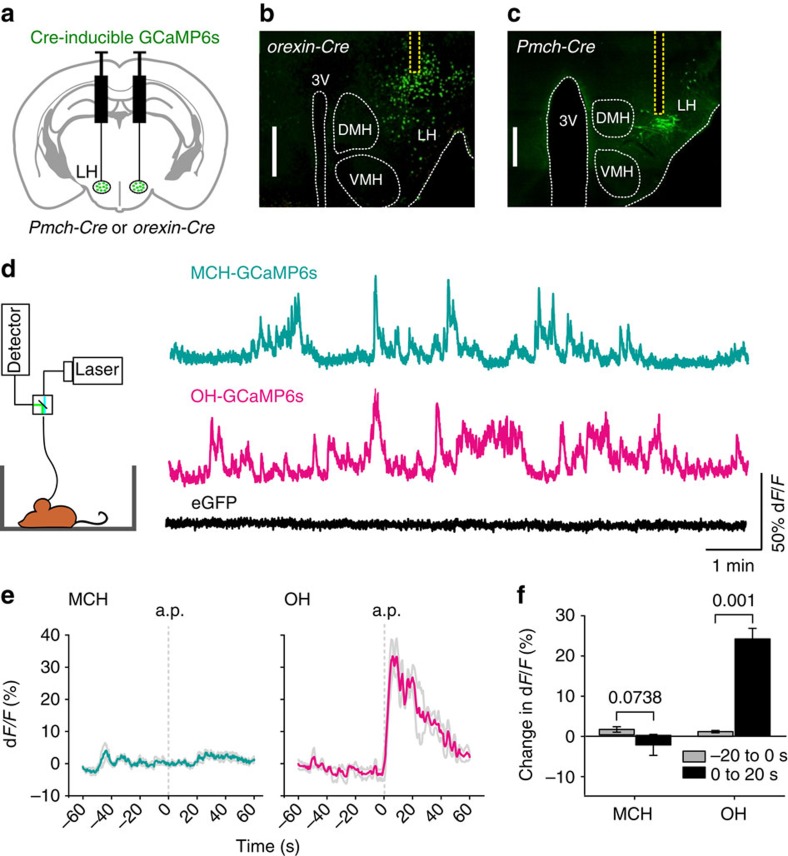
Optical recordings from MCH and OH cells in awake freely moving mice (**a**) Scheme for targeting GCaMP6s expression. (**b**,**c**) Coronal brain slices from *Pmch-Cre* and *orexin-Cre* mice showing fibre locations (yellow dashed lines) and injection sites. DMH, dorsomedial hypothalamus; LH, lateral hypothalamus; 3V, third ventricle; VMH, ventromedial hypothalamus. Representative images of *n*=6 brains per group. Scale bars, 500 μm. (**d**) Left, scheme for fibre photometry. Right, calcium signals during cage exploration from mice expressing GCaMP6s in MCH neurons or GCaMP6s or eGFP in OH neurons (representative examples of *n*=4, 4, and 3 mice, respectively). (**e**) Calcium signals from mice expressing GCaMP6s in MCH and OH cells, aligned to application of tail air-puff (a.p.). Turquoise or magenta lines indicate means, grey lines indicate s.e.m., *n*=4 mice per group. (**f**) Quantification of data in **e**. Changes in fluorescence (means±s.e.m., relative to values at −60 s) that occurred before a.p. (−20 to 0 s) and after the a.p. (0 to 20 s). Numbers above bars are *P* values from paired *t*-tests (MCH network: *t*, d.f.=0.367, 3; OH network *t*, d.f.=13.169, 3; *n*=4 mice per group).

To resolve MCH and OH network modulation at the time-scale of sensory processing, we utilized recent advances in fibre photometry^20^,^21^,^22^. We expressed Cre-dependent GCaMP6s calcium indicator in *Pmch-Cre* or *orexin-Cre* transgenic mice, and collected GCaMP6s fluorescence through a fiberoptic probe placed above the LH (Fig. 1a–c and Supplementary Fig. 1; see the Methods for details). This revealed activity transients in both OH- and MCH-GCaMP6s mice (Fig. 1d). These transients (≈10–50% change in fluorescence) resembled those recently recorded with similar methods in other deep networks^20^,^21^,^22^. Such transients are thought to represent synchronized population activity of GCaMP-expressing neurons^20^,^21^,^22^. Control experiments indicated that these activity fluctuations were not artefacts (for example, from movement). First, a brief somatosensory stimulus (tail air-puff) activated the OH network (as expected from responses of single OH cells^16^) but not the MCH network (Fig. 1e,f), and the MCH and OH signals were not strongly associated with movement (Supplementary Fig. 2a,b). This shows that the GCaMP6s activity transients are not general perturbation artefacts. Second, a non-activity-dependent probe (enhanced green fluorescent protein, eGFP) expressed in OH or MCH cells did not display GCaMP6s-comparable fluctuations in freely moving mice (Fig. 1d and Supplementary Fig. 3a–c), indicating that the fluctuations were related to neural activity. Together, these results demonstrate that both OH and MCH populations display rapidly modulated activity patterns in awake freely moving mice.

### Context-dependent activation of MCH cells

**Figure 2:**
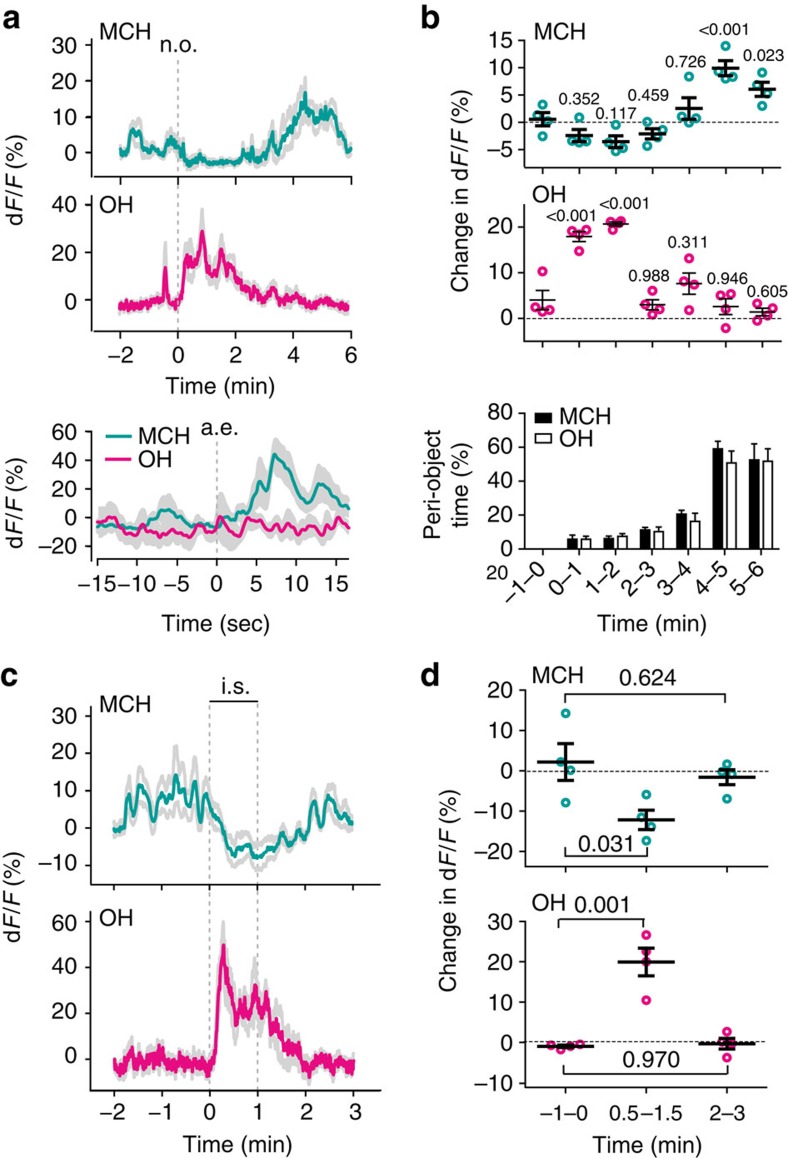
Differential modulation of MCH and OH cells by sensory experiences. (**a**) Calcium signals from mice expressing GCaMP6s in MCH or OH cells aligned to novel object (n.o.) presentation (top and middle plots) or to the time of object area entry (a.e.; bottom plot). Turquoise or magenta lines are means, grey lines are s.e.m., *n*=4 mice per group. (**b**) Binned quantification of data in **a** (top plots) and object area entry (bottom plot). Changes in fluorescence (means±s.e.m., black) and individual values, relative to values at −2 min, before and after n.o. appearance (at indicated times). Numbers above data are *P* values of Dunnett's multiple comparisons tests following one-way repeated measures analyses of variance (ANOVAs; MCH network: F(6,18)=16.67, *P*<0.0001; OH network: F(6,18)=31.79, *P*<0.0001). *n*=4 mice per group. (**c**) Calcium signals from mice expressing GCaMP6s in MCH or OH cells aligned to immobilization stress (i.s.) applied for 1 min. Turquoise or magenta lines are means, grey lines are s.e.m., *n*=4 mice per group. (**d**) Quantification of data in **c**. Changes in fluorescence (means±s.e.m., relative to values at −2 min, before, during and after i.s. (at indicated times). Numbers above data are *P* values of Dunnett's multiple comparisons tests following one-way repeated measures ANOVAs (MCH network: F(2,6)=5.7, *P*=0.041; OH network: F(2, 6)=27.77, *P*=0.0009). *n*=4 mice per group.

MCH cell inactivation disrupts appropriate coupling of a sensory experience to action selection^11^. This suggests that MCH cell activity may be linked to sensory evaluation. Such evaluation can occur on exposure to novel objects^23^,^24^. We found that MCH-GCaMP6s cell activity increased when novel objects (Fig. 2a,b), but not a familiar object (Supplementary Fig. 3d,e), were placed into the cage. This activation was not seen in control experiments with MCH-GFP mice (Supplementary Fig. 3b). The increased MCH-GCaMP6s signal was associated with times of object area entry (Fig. 2a,b and Supplementary Fig. 2c). Novelty has been proposed to elicit transient fear/stress followed by exploration^23^. OH cell activity has been proposed to represent stress states^25^. Indeed, upon novel object appearance, the OH cell activity became rapidly and significantly elevated (Fig. 2a,b; such changes were not seen in control OH-GFP mice, Supplementary Fig. 3b). The OH-GCaMP6s signal was greater within ≈2 min of novel object appearance when few object area entries occurred, than at the later time when more entries occurred (Fig. 2a,b and Supplementary Fig. 2c). Similarly timed, but smaller, changes in OH cell activity occurred after presentation of a familiar object (Supplementary Fig. 3d,e). Together, these data suggest that novel objects elicit both MCH and OH signals, but in a temporal sequence: first transient OH signals correlating with object avoidance, then MCH signals correlating with object exploration.

### Context-dependent inactivation of MCH cells

The temporal segregation of the MCH and OH signals *in vivo* is consistent with *in vitro* data showing that OH cells inhibit MCH cells via local microcircuits^19^. If such circuits operated *in vivo*, there should be a fall in MCH cell activity when the OH cell activity increases. This fall was not clearly apparent in Figs 1e,f and 2a,b. However, in these experiments, the baseline MCH cell activity was already low. Therefore, we re-examined MCH cell responses to a stimulus that activates OH cells, when MCH cell activity was elevated, which we found to be several minutes after novel object exposure (Fig. 2a). To create an OH-activating stimulus distinct from novel object, in these experiments we used acute physical immobilization, an established paradigm for eliciting stress responses in the mouse brain^26^,^27^. We found that acute immobilization stress (see the Methods for details) robustly and reversibly excited OH-GCaMP6s cells (Fig. 2c,d) but not OH-GFP control cells (Supplementary Fig. 3c). The same stimulus suppressed the activity of MCH-GCaMP6s but not control MCH-GFP cells (Fig. 2c,d and Supplementary Fig. 3c), a response significantly different from the OH cell response (during the 0.5–1.5 min after immobilization onset, fluorescence change relative to baseline at −2 min was −12.1±2.4% for MCH cells and 19.9±3.4% for OH cells; *P*=0.0003, two-tailed unpaired *t*-test; *t*, d.f.=7.652, 6; *n*=4 mice per group). These data further reveal differential modulation of MCH and OH networks on behaviourally relevant timescales across different sensory contexts.

### Brain-wide mapping of direct inputs to MCH and OH neurons

**Figure 3:**
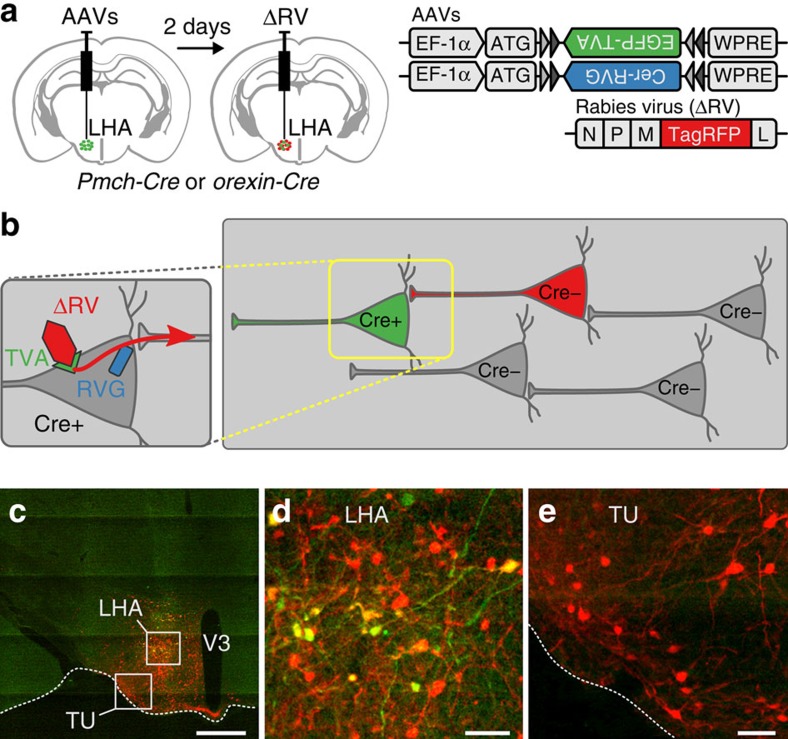
Strategy for genetically specified tracing of monosynaptic inputs to LH cells. (**a**) Scheme for targeting monosynaptic retrograde tracing components (Cre-dependent RVG and TVA, followed by rabies ΔRV than can only infect cells expressing TVA). (**b**) Scheme for monosynaptic retrograde tracing processes. Monosynaptic input neurons to Cre-positive cells (green) become selectively labelled with RFP (red), because the rabies-RFP (ΔRV) can only cross synapses if the infected cell contains the Cre-dependent RVG (zoom). (**c**) An example of a coronal brain section from a *Pmch-Cre* mouse expressing fluorescent proteins delivered by viral vectors as indicated in **a**,**b**. MCH neurons (green, yellow) and their direct input neurons (red), scale bar, 1 mm. Representative example from six brains. (**d**) Higher magnification image from **c** (LHA), scale bar, 200 μm. (**e**) Higher magnification image from **c** (TU), scale bar 200 μm.

**Figure 4:**
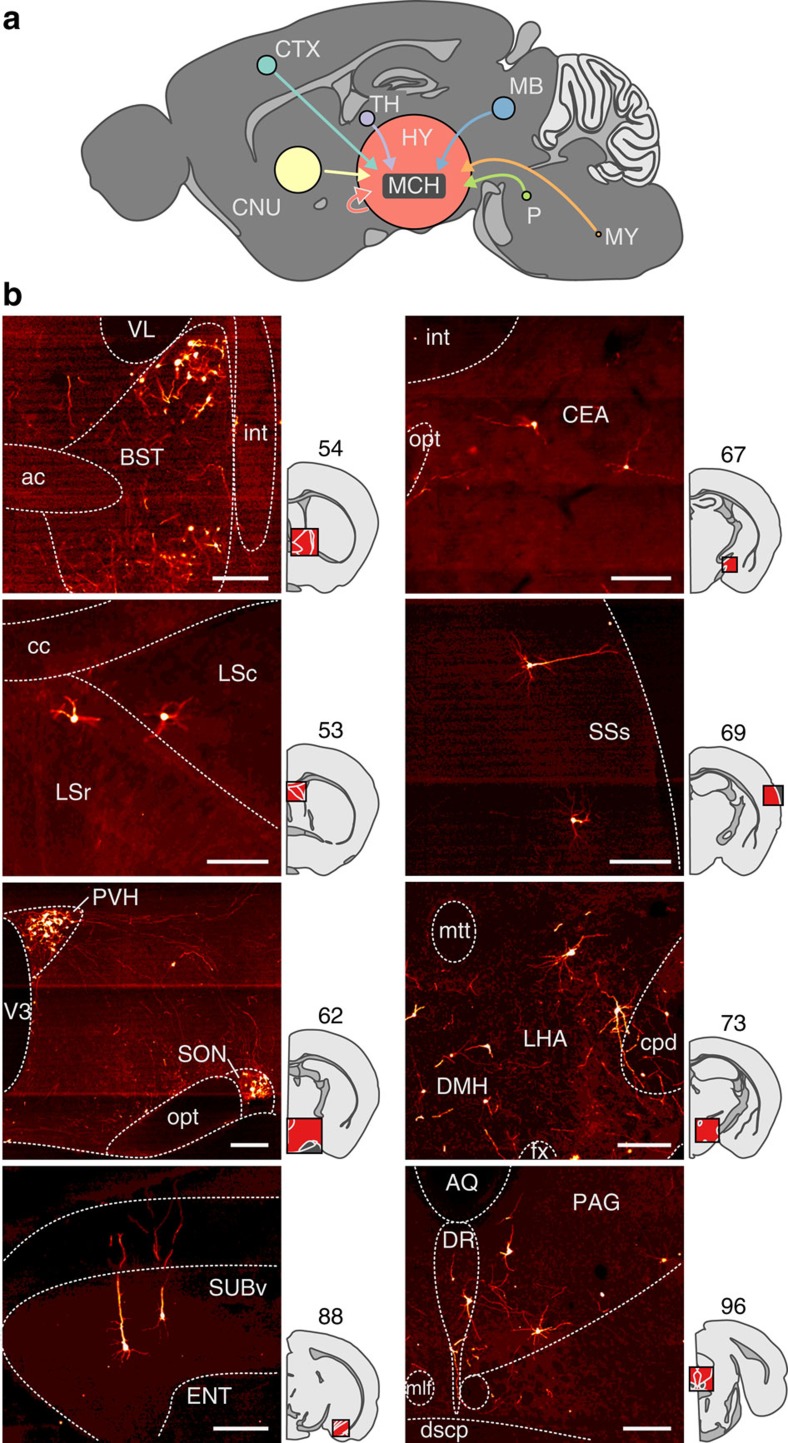
Coarse-grained whole-brain mapping of neural inputs to MCH cells. (**a**) Scheme showing relative contributions of seven large-scale areas to MCH cell input. The area of each circle represents the proportion of total whole-brain inputs (see the Results for details). Area names are based on the Allen Atlas: CNU, cerebral nuclei; CTX, cortex; HY, hypothalamus; MB, midbrain; MY, medulla; P, pons; TH, thalamus. (**b**) Examples of morphologies of neurons sending direct inputs to MCH neurons in different brain regions. The numbers above slice schematics correspond to image numbers in the Allen Atlas. Scale bars, 200 μm. Representative examples of *n*=6 brains. ac, anterior commissure; AQ, cerebral aqueduct; BST, bed nuclei of the stria terminalis; cc, corpus callosum; CEA, central amygdalar nucleus; cpd, cerebal peduncle; dscp, superior cerebellar peduncle decussation; DMH, dorsomedial nucleus of the hypothalamus; DR, dorsal nucleus raphe; ENT, entorhinal area; fx, columns of the fornix; int, internal capsule; LHA, lateral hypothalamic area; LSc, lateral septal nucleus, caudal part; LSr, lateral septal nucleus, rostral part; mlf, medial longitudinal fascicle; mtt, mammilothalmic tract; opt, optic tract; PAG, periaqueductal grey; PVH, paraventricular hypothalamic nucleus; SON, supraoptic nucleus; SSs, supplemental somatosensory area; SUBv, subiculum, ventral part; V3, third ventricle; VL, lateral ventricle.

The rapid MCH cell activity fluctuations *in vivo* (Fig. 1d) are unlikely to be caused by the (typically much slower) dynamics of circulating neuromodulators. To probe pathways for rapid control of MCH cells, we investigated neurons that provide direct inputs to MCH cells. To achieve this, we used a modified rabies virus tagged with red fluorescent protein (ΔRV-RFP, see the Methods for details). This virus only infects cells expressing the TVA receptor, and only crosses synapses if the infected cell expresses the rabies virus glycoprotein RVG^28^ (Fig. 3a,b). The infection and expression of ΔRV-RFP, therefore, occurs only in neurons expressing TVA+RVG and in their monosynaptic input neurons. We targeted TVA-eGFP and RVG-Cerulean to MCH or OH neurons by injecting Cre-inducible viral vectors coding for these proteins into the LH of the *Pmch-Cre* or *orexin-Cre* transgenic mice. Subsequent LH injection of ΔRV-RFP thus enabled spectral separation of MCH neurons or OH neurons (eGFP+) from distinct neurons that innervate them monosynaptically (RFP+/eGFP−). Several days after the viral injections (see the Methods for details), we observed networks of RFP+/eGFP− input neurons in both the hypothalamus and remote extra-hypothalamic regions (Figs 3c and 4b and Supplementary Fig. 4a,b). Our subsequent analyses focused on MCH cell inputs, as this information is still lacking. For comparison, we supplemented these results with more limited data on monosynaptic input to OH cells, which was previously estimated with polysynaptic or non-cell-specific monosynaptic tracers^29^,^30^.

**Figure 5:**
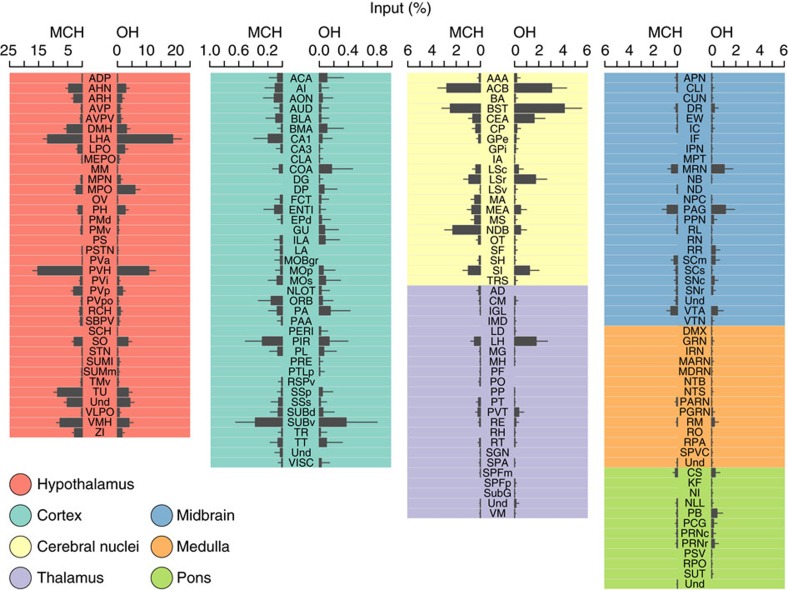
Fine-grained whole-brain mapping of neural inputs to MCH and OH cells. High-resolution quantification of monosynaptic inputs to MCH and OH neurons across 170 brain areas. Data are means±s.e.m. of cell percentages found per area in three *orexin-Cre* brains and six *Pmch-Cre* brains. ‘Undefined' refers to cells located outside anatomically named areas of the Allen Atlas. The anatomical acronyms are based on the Allen Atlas, and are fully defined in Supplementary Fig. 5.

To quantify and compare the brain-wide inter-areal distributions of MCH and OH cell inputs, we used fluorescent Hoechst staining to identify anatomical areas based on the Allen Brain Atlas for the mouse brain^31^. The location of each neuron was defined as an anatomical coordinate allocated to one of seven large-scale brain areas (Fig. 4a), and one of their subareas (Fig. 5 and Supplementary Fig. 5). To correct for the inter-animal variability in the number of labelled cells, we normalized input cell number in each region to the total input cell number in each brain (Fig. 5), or alternatively, also to the volumes of brain areas (Supplementary Fig. 5). The resulting global input maps revealed brain-wide distributions, and broad areal symmetries, of inputs to MCH and OH neurons (Fig. 5 and Supplementary Fig. 5). The greatest numbers of input neurons for MCH cells were found in hypothalamic networks (Fig. 5 and Supplementary Fig. 5), including hypothalamic neurons expressing peptides linked to emotional bonding, and energy and fluid homeostasis, such as oxytocin and vasopressin in the paraventricular hypothalamic nucleus, and proopiomelanocortin in the hypothalamic arcuate nucleus (Supplementary Fig. 4C). Two especially striking hotspots of MCH input neurons corresponded to hypothalamic paraventricular and supraoptic nuclei (Figs 4b and 5 and Supplementary Fig 4), followed by clusters of input cells in the bed nucleus of the stria terminalis (BST) and the amygdala (Figs 4b and 5 and Supplementary Fig. 5).

In terms of input percentages from large-scale, extrahypothalamic brain areas, the rank order for both MCH neurons and OH neurons was: cerebral nuclei>thalamus/midbrain>brainstem/cortex (Fig. 5 and Supplementary Fig. 5). Within most of these areas, the distribution of input hotspots was highly non-uniform. For example, among 36 hypothalamic subregions where we found input neurons, most inputs came from the paraventricular nucleus and the LH itself, followed by the medial preoptic area (Fig. 5 and Supplementary Fig. 5). Among the 21 cerebral nuclei containing input neurons, most inputs came from BST, nucleus accumbens, lateral septum and diagonal band nucleus (Fig. 5 and Supplementary Fig. 5). Among 25 midbrain areas containing input neurons, most inputs came from ventral tegmental area, midbrain reticular nucleus, periaqueductal gray and rostral linear nucleus raphe; whereas among 39 cortical areas containing input neurons, most input cells were seen in the ventral subiculum and posterior amygdalar nucleus (Fig. 5 and Supplementary Fig. 5).

Overall, these results demonstrate that MCH cells, as well as OH cells, serve as a convergence point of diverse direct inputs, distributed brain-wide, and likely carrying different types of information to be integrated in the LH.

### MCH cell inhibition by VGAT neurons of amygdala and BST

**Figure 6:**
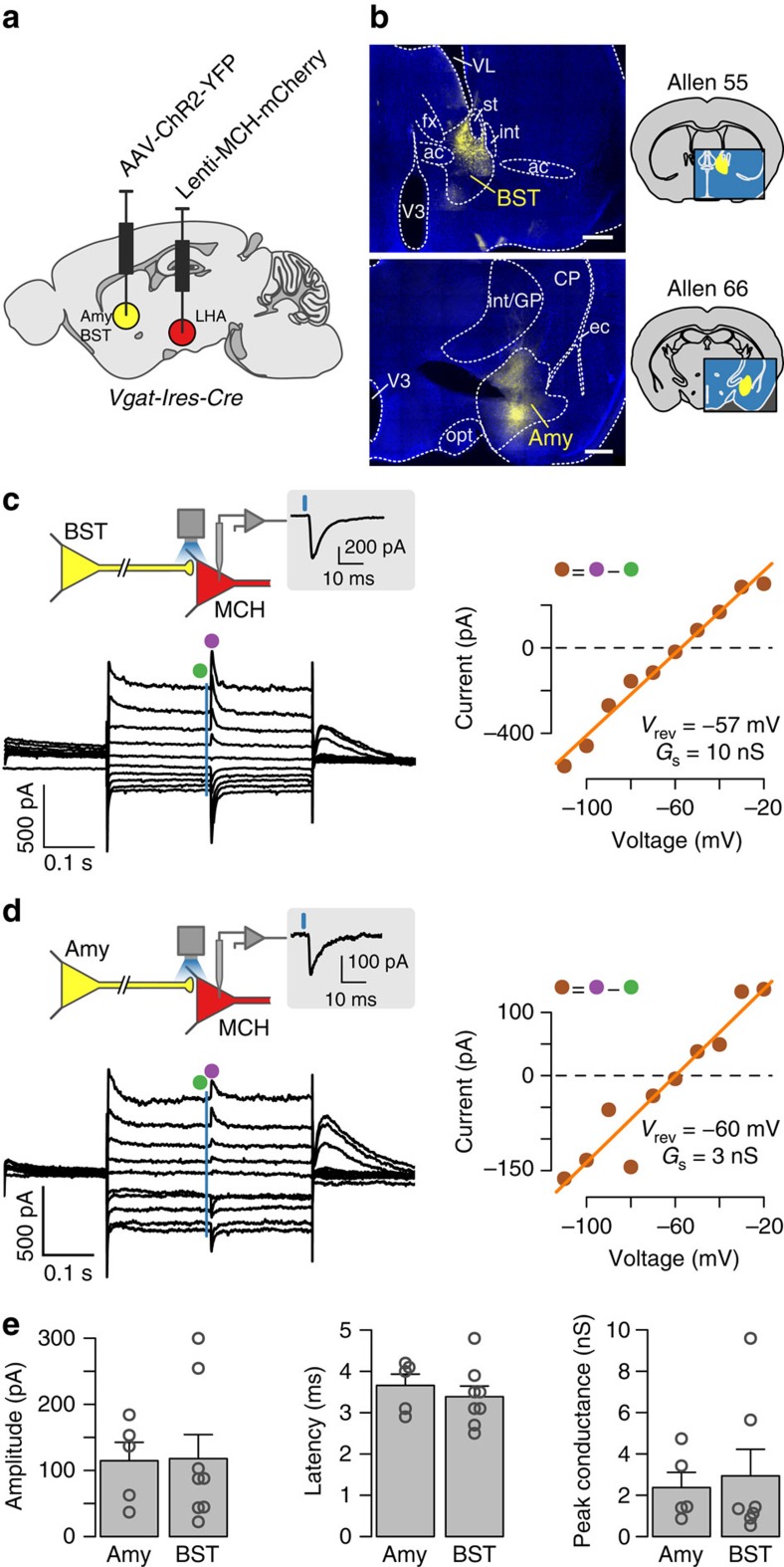
Functional inputs to MCH neurons from the amygdala (Amy) and bed nuclei of the stria terminalis (BST). (**a**,**b**) Scheme for targeting ChR2-eYFP and mCherry. Expression of ChR2-eYFP in the BST and Amy. Scale bars, 500 μm. Representative examples of four brains per each injection site. CP, caudoputamen; GP, globus pallidus; V3, third ventricle; VL, lateral ventricle; ac, anterior commissure; ec, external capsule; fx, columns of the fornix; int, internal capsule; opt, optic tract; st, stria terminalis. Image numbers from the Allen Atlas are given above brain sections. (**c**) Effect of BST_VGAT_ axon photostimulation (blue vertical line) on MCH neurons. Postsynaptic current was measured at different holding potentials. Representative example of 8 connected cells (8/19 cells were connected). (**d**) Effect of amygdala_VGAT_ axon photostimulation (blue vertical line) on MCH neurons at different holding potentials Postsynaptic current was measured at different holding potentials. Representative example of 5 connected cells (5/13 cells were connected). (**e**) Properties of photostimulation-induced postsynaptic currents. Amplitude and latency were measured at a holding potential of −10 mV. Each point represents a cell, bars are means±s.e.m. No significant differences between Amy and BST were found in connection amplitudes (Amy=114.7±27.9, *n*=5; BST=117.9±36.3 pA, *n*=8, *P*=0.833, Wilcoxon rank sum test), latencies (Amy=3.66±0.27, *n*=5; BST=3.39±0.26 ms, *n*=8, *P*=0.485, Student's *t*-test) or conductances (Amy=2.37±0.73, *n*=5; BST 2.94±1.29 nS, *n*=7, *P*=0.755, Wilcoxon rank sum test).

We next sought a proof-of-concept validation that our anatomical mapping corresponds to functional interactions. Our data suggested that stress antagonises MCH network signals (Fig. 2). Stress and anxiety also antagonize key functions attributed to MCH neurons, such as sleep, appropriate food choice, weight gain and synaptic plasticity. We noted that, among stress-implicated areas projecting to MCH cells, two hotspots were especially prominent: the amygdala and the BST. These areas contain GABAergic inhibitory neurons expressing VGAT^32^,^33^. Therefore, we tested whether these VGAT neurons directly inhibit MCH cells. To achieve this, we paired optogenetic stimulation of VGAT projections neurons from these regions with electrophysiological recordings from identified MCH neurons in brain slices. To enable optical stimulation of the VGAT cells, we injected a Cre-inducible Channelrhodopsin-2 (ChR2-eYFP) into the BST or amygdala of *Vgat-Ires-Cre* transgenic mice (Fig. 6a,b). To identify MCH neurons for recordings, in the same brains, we also injected, into the LH, a lentiviral construct coding for an mCherry fluorescent tag driven by the MCH promoter^19^ (Fig. 6a and Supplementary Fig. 6).

Photostimulation of ChR2-containing axon terminals from VGAT neurons of the BST or amygdala produced robust synaptic responses in 42% and 38% of MCH neurons, respectively (Fig. 6c,d). For both projections, the fast synaptic currents triggered in MCH neurons by the optical stimulation changed polarity near the equilibrium potential for chloride, as expected from ionotropic GABA receptors (Fig. 6c,d). The latencies of amygdala_GABA_→LH_MCH_ and BST_GABA_→LH_MCH_ inputs were short, ≈3–4 ms on average (Fig. 6e), consistent with direct connectivity. The amplitudes and conductances of amydgala_VGAT_→LH_MCH_ and BST_VGAT_→LH_MCH_ functional connections were similar (Fig. 6e; statistics are given in the figure legend). These data provide proof-of-concept evidence that anatomical maps highlighted by our retrograde tracing correspond to functional circuits, and demonstrate that VGAT cells in the BST and amygdala exert rapid, direct inhibitory control over MCH neurons.

## Discussion

Despite the importance of the MCH network for the control of brain state, decision-making, and energy balance, the nature of its *in vivo* dynamics has been poorly understood. Our results define several natural factors that rapidly influence MCH network activity. To the best of our knowledge, this study provides the first evidence that the MCH network is rapidly, differentially modulated during different awake experiences, and is anatomically and functionally wired to receive signals from diverse and distributed neural constellations. A key unresolved question in the field has been whether, in the brain *in vivo*, MCH and OH signals co-activate (as implied by *in vitro* single-cell recordings of OH-induced MCH cell activation^18^) or alternate (as implied by *in vitro* analysis of network interactions^19^). Our *in vivo* results suggest that the timing of MCH cell signals during wakefulness is largely distinct from that of OH cell signals.

Our findings advance and complement existing models of the LH, a classic hub for orchestrating vital behaviours^34^. MCH neurons innervate key memory-related structures, including the hippocampus and septum where MCH receptors promote synaptic strengthening^14^,^35^. MCH cell activity can encode the nutrient value of extracellular space^11^,^36^. If a sensory event, such as tasting food, coincides with experimental (optogenetic) activation of MCH cells, it evokes greater neurochemical signals of reward, and becomes more likely to be selected again^11^. Such data have been conceptualized into models where MCH cell activity modulates behaviour based on circulating hormones and nutrients. However, MCH cell activity has hitherto been observed only during sleep^15^. Without the knowledge of whether MCH cell activity is present and modulated during wakefulness, it is not possible to assemble a coherent understanding of the role of MCH cells in the brain function. Our data supply this knowledge by providing evidence of physiological modulation of MCH cell activity during wakefulness. Pioneering single-cell recordings performed previously on MCH cells showed them to be active during sleep, but silent during wakefulness under physical restraint^15^. Our new data now suggest that MCH cell activation can also occur during wakefulness, for example, under conditions that could be described as ‘non-stressful novelty'. The previous demonstration of high-selectivity of Cre and GCaMP6s targeting to MCH cells in the MCH-Cre line we used for photometry^19^ supports the idea that the GCaMP6s signals we recorded predominantly originated from the MCH cells themselves.

We could not precisely infer cell firing rates from the GCaMP6s signals *in vivo*. However, our *in vitro* experiments correlating firing frequency and GCaMP6s fluorescence in MCH cells show that a 10-Hz increase in activity corresponds to ≈10–40% increase in fluorescence (median ≈14%, Supplementary Fig. 1). Based on this, the fluorescence changes in MCH cells *in vivo* (≈10–50% peak, ≈15% average, Figs 1d and 2a) may correspond to firing changes in the order of 10–40 Hz. This is within the physiological firing spectrum of MCH cells *in vivo* (≈0–60 Hz instantaneous firing, see Supplementary Fig. 1B in Hassani *et al.*^15^).

We demonstrate that VGAT neurons in the amygdala and BST inhibit MCH neurons. However, it is likely that additional neural inputs communicate stress and other states to MCH cells. Indeed, our connectivity mapping suggests widely distributed, degenerate (many-to-one) neural input architectures for both MCH and OH cells. For the MCH network, degenerate input processing was implied by both structure (multiple inputs converging onto MCH neurons, Figs 4 and 5) and function (different inputs having similar functional outcomes, Fig. 6). There is much evidence that the retrograde tracing method we used is monosynaptically restricted^37^. Although host cell death may theoretically release virus into the extracellular space, we are not aware of any evidence that such virus would be competent-enough to cause significant infection of surrounding cells and consequent non-specific retrograde labelling. Nevertheless, because of this theoretical caveat, it was important to examine whether the neural connections identified by the rabies tracing correspond to functional inputs. Our functional optogenetic validation of key connections identified by the retrograde tracing (Fig. 6) is an important confirmation of the value of the rabies methods for finding sources of direct neural inputs to MCH cells. It is currently unclear (and cannot be inferred from the techniques used in this study) whether the same input neurons innervate both MCH and OH cells. Dissecting the contributions of the many input nodes identified here would require specific silencing of separate circuits during behaviour *in vivo*, while recording MCH cell representations of sensorimotor states.

In summary, our data demonstrate that MCH neurons generate conditional population bursts during awake sensorimotor processing. We propose that the fluctuations in MCH cell activity are the result of integrating signals from many brain areas, including inhibitory signals transmitting stress-related information. The distributed and heterogeneous input circuitry revealed by our connectivity mapping opens up the field for further conceptualization of data streams that enable LH neurons to orchestrate activity in their brain-wide projection fields.

## Methods

### Gene transfer

All animal procedures performed in this study were approved by the UK government (Home Office) and by Institutional Animal Welfare Ethical Review Panel. Adult male mice (5–12 weeks old) were used. Animals were single-housed and kept on a standard 12-h light–dark cycle (lights on at 0700, hours) and on standard mouse chow and water *ad libitum*. For calcium imaging, adeno-associated viral vectors (AAVs) carrying cre-dependent GCaMP6s (rAAV9.CAG.Flex.GCaMP6s.WPRE.SV40; Penn Vector Core) were bilaterally stereotaxically injected into the LH of *Pmch-Cre* or *orexin-Cre* mice. Three 50 nl injections of the GCaMP6s virus were delivered at: 1.35 mm caudal from bregma; ±0.9 mm lateral from midline; and 5.30, 5.20 and 5.10 mm ventral from brain surface. In the control ‘eGFP' mice (Fig. 1 and Supplementary Fig. 3), a vector delivering Cre-dependent eGFP (AAV1.CAG.Flex.eGFP; UNC Vector Core) was injected in the LH of appropriate Cre mice.

For cell-type-specific retrograde tracing, 50–60 nl of AAV2/1-EF1a-Flex-C-RVG (Addgene, 49101) and AAV2/1-EF1a-Flex-eGFP-TVA (Addgene, 26198, Wall *et al.*^28^) were stereotaxically microinjected^38^ into the LH of *Pmch-cre* or *orexin-Cre* mice. The LH injection coordinates were: −1.35 mm caudal from bregma; 0.75–0.9 mm lateral from midline; 5.3–5.4 mm ventral from brain surface. Two days later, we injected at the same site 70 nl of envelope A pseudotyped SADB19 rabies virus expressing tagRFP in place of rabies glycoprotein (ΔRV-RFP^39^, prepared as in ref. 40). For optogenetics, 50–70 nl of rAAV2/1.EF1.DIO.hChR2(H134R)-YFP.WPRE.hGH (1.5 × 10^12^ gc ml^−1^; Addgene #20298; packaged at Vector Core, University of Pennsylvania) was microinjected into the BST or amygdala of *Vgat-Ires-Cre* transgenic mice. Injection coordinates were: amygdala (covering central and medial areas): 2.6 mm lateral from midline, 1.2 mm caudal from bregma, 4.7 mm ventral from the brain surface; BST: 1.0 mm lateral from midline, 0.14 mm rostral from bregma, 3.7 mm ventral from the brain surface. On the same day, 200 nl of a lentiviral vector that selectively expresses mCherry fluorescent tag in MCH neurons under the control of MCH promoter^18^ was injected into the LH; this produces specific labelling of MCH neurons with no disruption in their electrical properties^19^.

Three Cre-expressing transgenic mouse lines were used:: *Pmch-Cre* mice^19^,^41^, *Vgat-Ires-Cre* mice^42^ and *orexin-Cre* mice^38^,^43^. All transgenic mice bred in heterozygous-WT breeding pairs with C57BL/6 mice. *orexin-Cre* mice were a kind gift from Professor Takeshi Sakurai, and the other two lines were obtained from The Jackson Laboratory (stock numbers: 014099, 016962). Cre-independent (non-specific) viral expression was not detected after injecting cre-dependent viruses into the brains of Cre-negative C57BL/6 mice (*n*=3-6 mice for each virus). In the *Pmch-Cre* transgenic line we used for photometry, MCH is expressed in >99% of Cre-positive cells, whereas 85% of MCH cells contained Cre^19^.

### Fibre photometry

A fibre photometry setup was built following a general design of Gunaydin *et al.*^21^, with minor changes. A 473-nm pulsed laser (Becker & Hickl) was used as the excitation source. The excitation light was sent into a fibre-connectorized cube containing a dichroic mirror and GFP excitation and emission filters (Doric, FMC_GFP_FC), and from there into a patchchord (Doric, MFP_200/230/900-0.22_2m_FCM-MF) linked to a custom fiberoptic implant (Doric, MFC_200/260-0.37_50mm_MF2.5(7.5mm)_FLT) through a brass sleeve (Doric, SLEEVE_BR_2.5). The fluorescence output was fibre-coupled from the GFP cube to a photodetector (Becker & Hickl, HPM-CON-2). The analogue detector signal was then sent to an AD port of an amplifier (HEKA, EPC-10), low-pass filtered at 3 kHz, and digitized at 500 Hz. The signal was recorded using software provided with the amplifier (HEKA Patchmaster suite).

The fiberoptic implant was stereotaxically installed with the fibre tip above the LH, and fixed to the skull^9^. The LH location of fibre tips were verified post-recording by examining slices with visible fibre-tract; because most of these slices were damaged, intact slices that were closest to the fibre tip in the anterio-posterior plane were used for Fig. 1b,c, and the fibre tract was drawn. Before implantation, laser emission from the implant fibre tip was directly measured (X-Cite Optical Power Measurement System, Excelitas Technologies) and adjusted to 0.1 mW, to keep photoexcitation power similar across brains. Detector signals were converted to d*F*/*F* (%) values as follows: d*F*/*F*=100*(Fr–*F*)/*F*, where Fr is the raw signal and *F* is the trial median. Head velocity videoanalysis (Supplementary Fig. 2a,b) was performed with ANY-maze software.

To control for circadian factors, the experiments were performed either at the start of the dark phase (1900 hours to 2100 hours) or in the middle of the light phase (1400 hours to 1600 hours); no differences in the reported responses were noted between the two light phases. All recordings were performed when the animal was actively moving and not asleep. Mice were grouped according to genotypes and vectors injected into their brains, and all groups had similar composition based on sex (males).

### *In vivo* manipulation

In ‘air puff' experiments (Fig. 1e), mice were placed in a recording cage, left for 10 min, then a trial was performed, consisting of: 1 min baseline recording, a brief (<1 s) air puff to the tail (Airduster), 1 min recovery recording. In ‘novel object' experiments (Fig. 2a), mice were placed in a recording cage, left for 10 min, then a trial was performed, consisting of: 2 min baseline recording, introducing an object into the cage, then recording for 6 min. Three novel object trials per mouse were performed, each with a different novel object to prevent habituation. Peri-object time was defined as the time when the animal's nose was within a 2-cm perimeter from object edges. Plot of signals aligned to object area entry (Fig. 2a, bottom plot) was generated from data recorded >3 min after object appearance (when mice explored the object the most, Fig. 2b, bottom plot). In control experiments (Supplementary Fig. 3d), three trials per mouse were performed using a familiar object. Novel objects were: a pebble, a plastic lid, a wooden cube. Familiar object was a wooden cube previously kept in home cage overnight. Novel and familiar objects were similarly sized (∼2.5 × 2.5 cm). Novel and familiar trials were interleaved to avoid order effects, and for each mouse the trial frequency was once a day or less, to reduce habituation. In ‘immobilization' experiments (Fig. 2c), mice were placed in a recording cage, left for 10 min, presented with a novel object for 4 min, then a trial was performed, consisting of: 2 min baseline recording, 1 min restraint stress (holding the mouse inside the cage by clamping the head-fixed ferrule in place), 2 min recovery recording. For each mouse, three trials were performed and trial frequency was once a day or less, to reduce habituation. In all cases, mice were habituated to the recording cage, to diminish the effects of cage novelty.

### Histology and image analysis

The animals were perfused 10–11 days after ΔRV-RFP injection with PBS followed by 4% PFA, and their brain was extracted and placed in 30% sucrose (in PBS) for ∼48 h. The brains were then frozen and stored at −80 °C until needed. Coronal sections of 50-μm-thick were cut with a cryostat from olfactory bulb to brainstem. The sections were stored in cryprotectant at −20 °C until needed. Of these, one section every third was taken for immunohistochemistry, which was performed with anti-GFP (1:500, chicken IgY fraction, Invitrogen, A10262) and anti-tRFP (1:1000, rabbit, Evrogen, AB234) primary antibodies followed by anti-rabbit Alexa 555 (1:500, Invitrogen, A21428) and anti-chicken IgY DyLight 488 (1:500, Abcam, ab96947) secondary antibodies. The anti-tRFP antibody was validated by western blot analysis (http://www.evrogen.com/products/antibodies/AB-tRFP_Data_sheets.shtml). The anti-GFP staining specificity was validated by observing an absence of staining on the side of the LH contralateral to the side where the AAV2/1-EF1a-Flex-eGFP-TVA was (unilaterally) injected. Hoechst was used for counter-staining. Full-section images were captured semi-automatically with an Olympus VS120 scanning microscope (× 10 objective). The vast majority of input cells were seen on the side ipsilateral to the LH tracer injection. The locations of the labelled neurons and the brain areas were determined manually using custom-written software in Python (http://www.python.org), and subsequent analyses were done using R (R Core Team, 2014; http://www.R-project.org). Any custom software can be requested from the authors. For anatomical reference, we used the Allen Mouse Brain Atlas^31^ (version 2, http://mouse.brain-map.org/static/atlas).

Although the viruses were injected as similarly as possible across brains, it is possible that the penetrance varied between brains; we did not examine this by cell counts *post mortem* (because absolute counts may be distorted by stochastic host-cell death after rabies infection), but instead normalized values (fluorescence, total number of input cells) within each mouse.

### Brain slice optogenetics, electrophysiology and imaging

Whole-cell patch-clamp recordings and photostimulation in acute brain slices were carried out using standard methods^38^. Briefly, LH slices were prepared at least 8 weeks after virus injections. Living neurons containing fluorescent markers were visualized using an upright Olympus BX61WI microscope equipped with an oblique condenser and appropriate fluorescence filters. Excitation light for ChR2 (∼10 mW mm^−2^ at the specimen) was delivered onto recorded neurons and their surrounding ChR2-expressing axons via a × 40, 0.8 numerical aperture objective from a LAMBDA DG-5 fast beam switcher (Sutter) with a xenon lamp and ET470/40 nm band-pass filter. Note that transmitter release from ChR2 axons does not require them to be connected to cell bodies^44^. The extracellular solution consists of following components (in mM): 125 NaCl, 2.5 KCl, 1.2 NaH_2_PO_4_, 21 NaHCO_3_, 1 glucose, 2 MgCl_2_, 2 CaCl_2_. The pipette solution consists of following components (in mM): 120 K-Gluconate, 10 HEPES, 10 KCl, 1 EGTA, 2 MgCl_2_, 4 K_2_ATP, 1 Na_2_ATP. The chloride equilibrium potential was calculated to be −60.3 mV using Nernst equation. In whole-cell recordings, liquid junction potential was estimated to be 10 mV and subtracted from the measurements. Neurons were randomly sampled throughout the full extent of the LH, by choosing fluorescent MCH cells using an objective that blinded the investigator to exact intra-LH location of the cell due to its small field of view (a × 40 objective).

In Supplementary Fig. 1b–e, to investigate the relationship between GCaMP6s fluorescence and cell activity, we used *in vitro* cell-attached electrophysiology together with epifluorescence and video recording. MCH-cre cells were tagged with GCaMP6s and mCherry using the AAVs as described above, and their activity was recorded using the cell-attached configuration in the voltage-clamp mode^45^. Voltage steps of increasing magnitude were applied to the cell, and GCaMP6s signals were captured at 25 frames per second. using a DAGE-MTI camera, a xenon excitation lamp and standard eGFP filters, while recording the cell firing rate.

### Statistics

Statistical tests and descriptive statistics were performed as specified in the figure legends and the text. For parametric tests, the variables under consideration were assessed for normality using KS and Shapiro–Wilk normality tests, and variances were compared using Levene's and Brown–Forsythe tests. For non-significant test outcomes, a power calculation at a significance level (alpha) of 0.05 and with 80% power was used to decide if the sample size was powerful enough to detect small effects. Data analyses were performed using Origin Pro 2015, Prism 6 or R (http://www.R-project.org). *P* values smaller than 0.05 were considered significant.

## Additional information

**How to cite this article:** González, J. A. *et al.* Awake dynamics and brain-wide direct inputs of hypothalamic MCH and orexin networks. *Nat. Commun.* 7:11395 doi: 10.1038/ncomms11395 (2016).

## Supplementary Material

Supplementary Information Supplementary Figures 1-6
