# Supplementary material for: Awake dynamics and brain-wide direct inputs of hypothalamic MCH and orexin networks
Source: Nat Commun. 2016 Apr 22;7:11395. doi: 10.1038/ncomms11395 (PMC4844703; doi:10.1038/ncomms11395)
Supplement: Supplementary Information — Supplementary Figures 1-6 [file ncomms11395-s1.pdf]

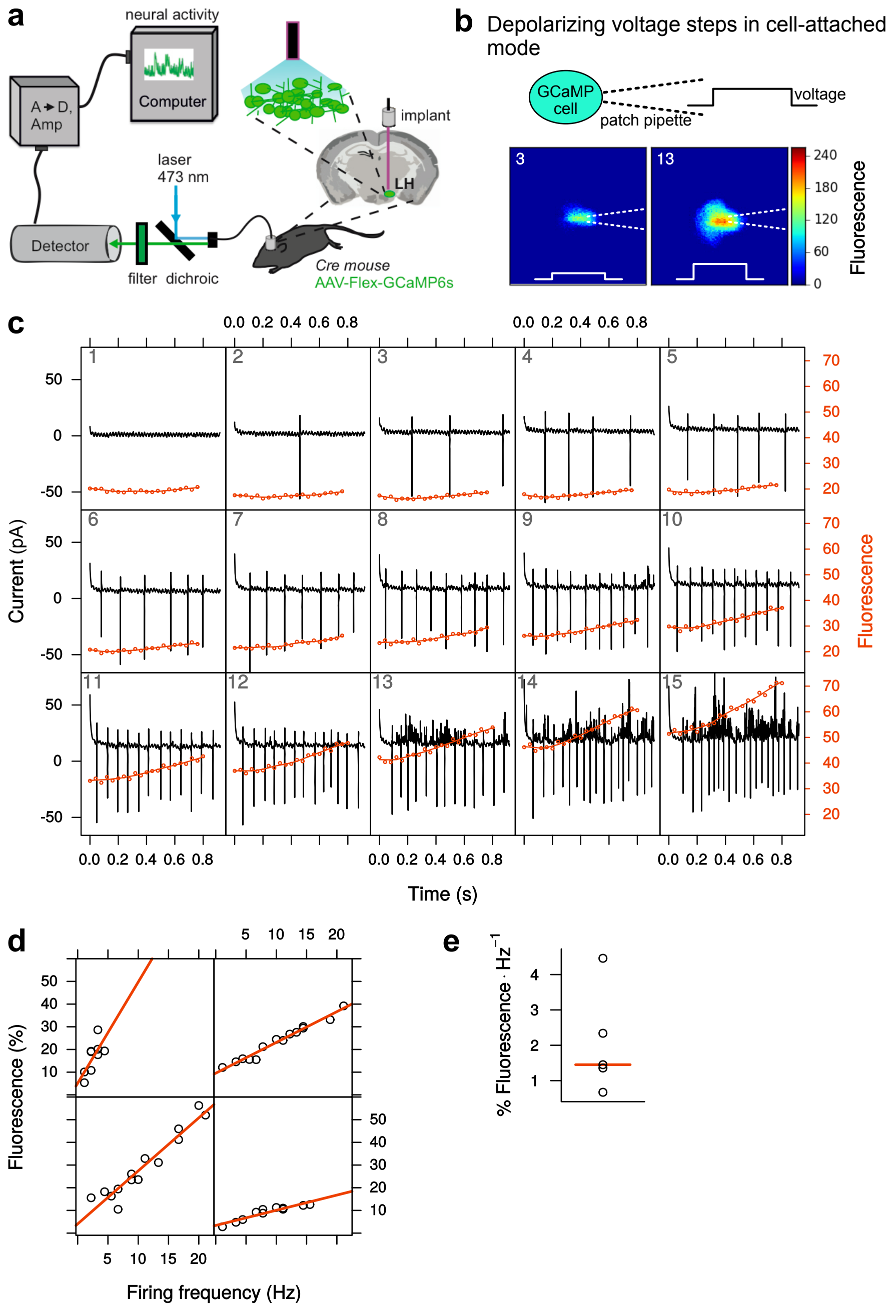

Supplementary Figure 1

Fibre photometry methodology and calcium recordings. (a) Detailed schematic of the *in vivo* fibre photometry set-up. **(b)** Top, schematic for recording GCaMP6s signals while varying and recording firing rate of individual neurons *in vitro* (see Methods). Bottom, example of two frames from a video recording from the cell illustrated in (c). Fluorescence is reported in raw pixel values and the position of the recording/stimulating pipette is indicated with dashed lines. The numbers correspond to sweeps in (c) (representative example of 5 MCH-GCaMP6s cells). **(c)** Typical example of simultaneous cell-attached and fluorescence recording from an MCH cell stimulated by depolarizing voltage steps of increasing intensity (representative example of 5 cells, see Methods). Fluorescence intensity here is average intensity (pixel values) of the recorded cell per video-frame. **(d)** Examples of the relationship between GCaMP6s signal and action potential firing in four different MCH-GCaMP6s cells. Percent fluorescence was calculated from raw signals such as that shown in (c) as $\Delta F/F_0 \times 100$, where F_0 is fluorescence at the beginning of the depolarizing pulse and ΔF is the difference in fluorescence between the end and the beginning of that same pulse. **(e)** Change in GCaMP6s fluorescence by firing frequency for 5 MCH-GCaMP6s cells, obtained from the slope of the linear fit of data such as those shown in (d). The median value (orange line) represents a 1.4% fluorescence increase for every 1 Hz increase in cell firing.

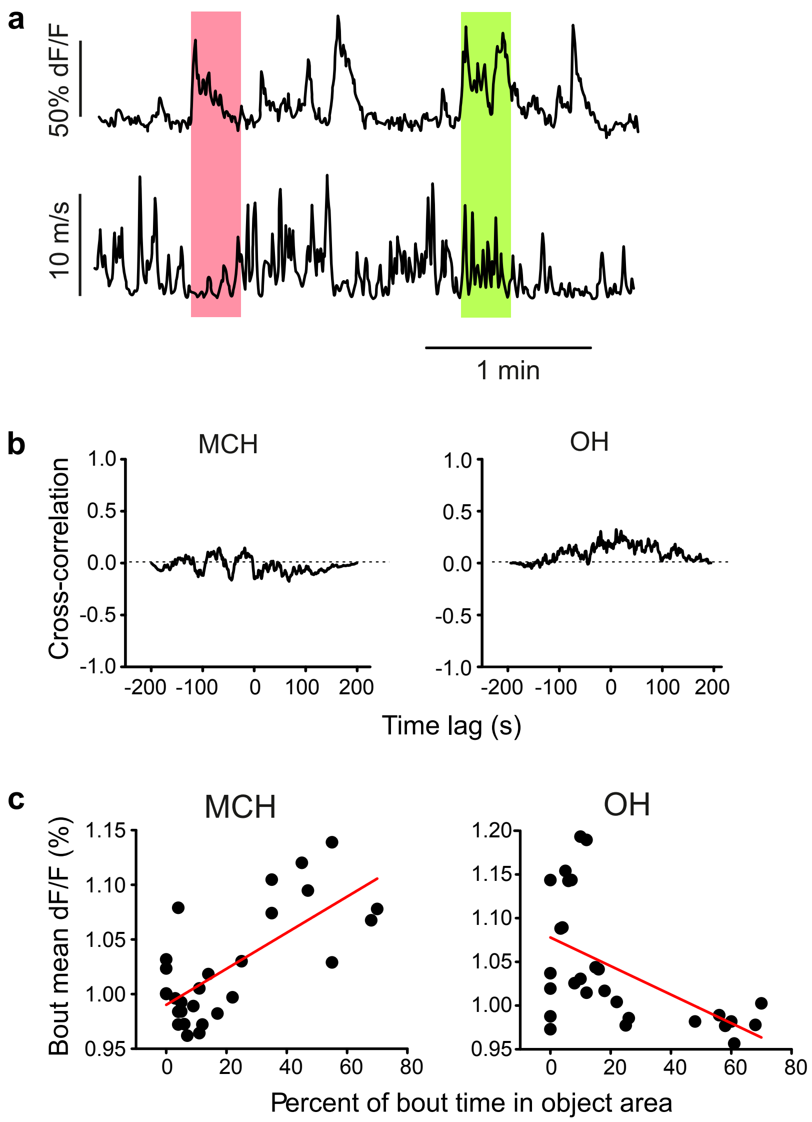

Supplementary Figure 2

Correlation between GCaMP6s signals and behaviour. (a) Recording of head velocity from a mouse expressing GCaMP6s in MCH neurons. Note that phasic activity bursts are seen during both low (red) and high (green) movement. **(b)** Cross-correlations of GCaMP6s signals and head velocity. **(c)** GCaMP6s signals aligned to novel object area entry (nose within 2 cm from object edges), in the experiment shown in Fig. 2a,b. The data are binned into one-minute bouts as indicated in Fig. 2b. Red lines are linear regression fits: Pearson's $r = 0.71$ (MCH) and -0.54 (OH).

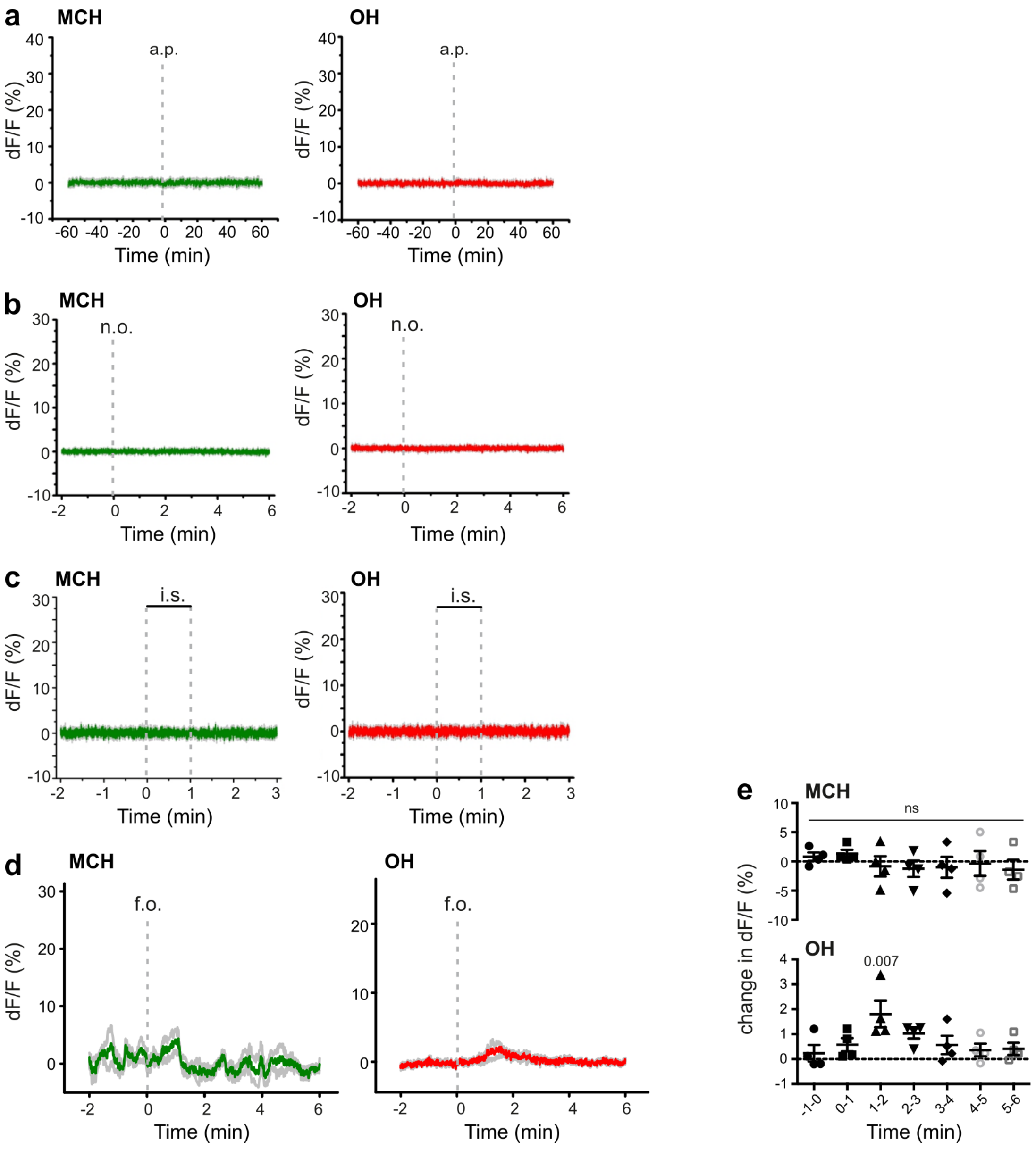

Supplementary Figure 3

Controls for photometry experiments. (a–c) Control experiments in mice expressing eGFP in MCH or OH neurons (see Methods), for results shown in main Fig. 1e (**a**; a.p., air puff), 2a (**b**; n.o., novel object), and 2c (**c**; i.s., immobilization stress). Green or red lines are means, grey lines are s.e.m., $n = 4$ mice in each group. **(d)** Calcium signals from mice expressing GCaMP6s in MCH or OH cells aligned to familiar object (f.o.) presentation. Green or red lines are means, grey lines are s.e.m., $n = 4$ mice in each group. **(e)** Quantification of data in (d). Changes in fluorescence (means $\pm$ s.e.m. and individual values, relative to values at -2 min, before and after f.o. appearance at indicated times). Number above bars is the only significant P value from Dunnet's multiple comparisons test following one-way repeated measures ANOVAs (MCH network: $F(6, 18) = 1.238$, $P = 0.333$; OH network: $F(6, 18) = 3.402$, $P = 0.0201$; $n = 4$ mice in each group). Non-significant values (ns) were $P \geq 0.05$.

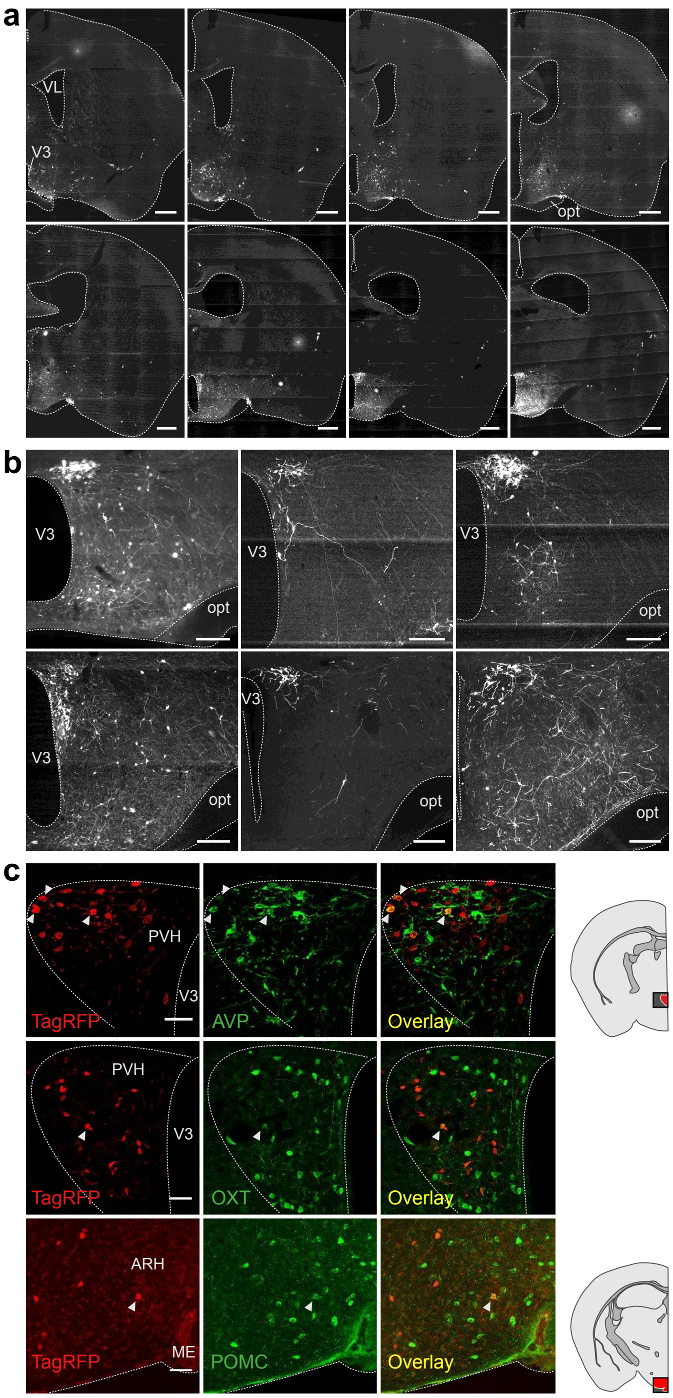

Supplementary Figure 4

Examples of input cells directly connected to MCH neurons. (a) Representative rostro-caudal sequence of brain sections from a *Pmch-cre* mouse (images 52–65 from the Allen Brain Atlas). RFP-expressing input cells are shown in white pseudo colour. Scale bars, 0.5 mm. **(b)** The paraventricular nucleus of 6 different *Pmch-cre* animals showing MCH-projecting cells (white pseudo colour). This was one of the densest MCH input hotspots. Scale bars, 200 µm. **(c)** Immunoreactivity for neuropeptides (green) in hypothalamic neurons (red) that provide direct inputs to MCH neurons. Scale bars, 200 µm. The antibodies were: vasopressin (AVP): primary PS41 mouse hybridoma 1:100 (ATCC CRL-1799, kind gift from Prof. David Murphy), secondary 1:500 donkey anti-mouse Alexa Fluor 647 (Life Technologies); Oxytocin (OXT): rabbit anti-oxytocin 1:1000 (Millipore, AB911) and donkey anti-rabbit Alexa Fluor 647 1:500 (Life Technologies); pro-opiomelanocortin (POMC): rabbit anti-POMC 1:1000 (Phoenix Pharmaceuticals, H-029-30) and donkey anti-rabbit Alexa Fluor 647 (as above). ARH, arcuate hypothalamic nucleus; ME, median eminence; opt, optic tract; PVH, paraventricular hypothalamic nucleus; V3, third ventricle; VL, lateral ventricle.

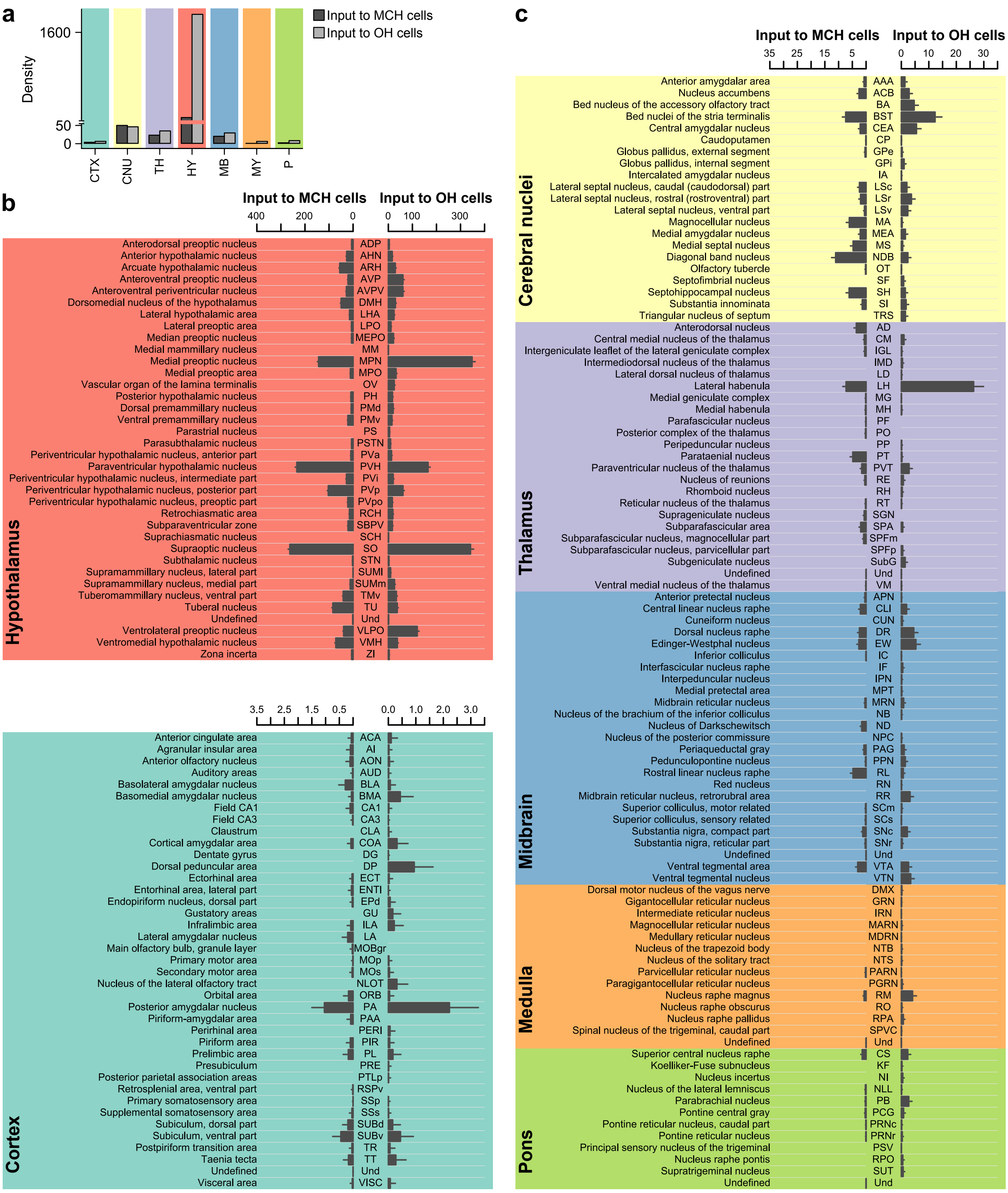

Supplementary Figure 5

Input densities. (a) Coarse-grained inter-areal distribution for the large areas specified in (b) and (c). **(b, c)** Fine-grained inter-areal distribution for smaller sub-areas. The relative volume of each brain area was calculated by adding the structure area across all images in the Allen Mouse Brain Atlas and dividing this volume by the overall brain volume. To obtain the relative cell density presented in the figure, the number of cells counted (Fig. 5) was divided by the relative volume of the corresponding brain structure.

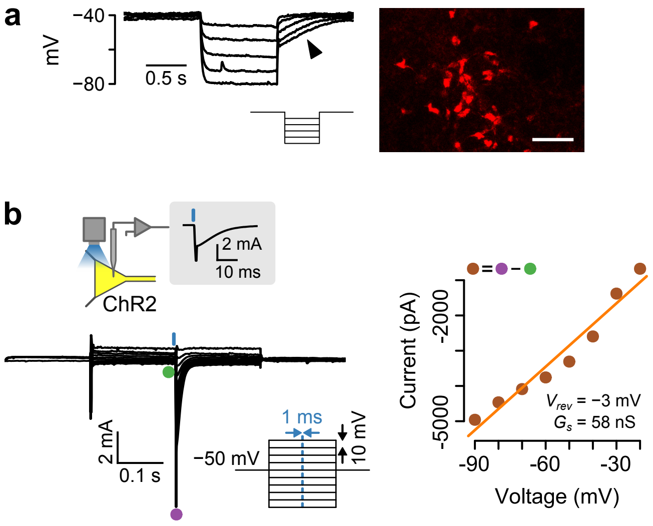

Supplementary Figure 6

MCH neuron identification and VGAT neuron manipulation for optogenetics. (a) Left, a current-clamp recording from an MCH cell identified by targeted mCherry expression, showing a large A-current-mediated rebound hyperpolarization (arrowhead) after step injections of hyperpolarizing current; the injection schematic is shown below the trace. Representative example of 32 neurons. Right, a confocal image of MCH-mCherry cells in the lateral hypothalamus in a brain slice. Scale bar, 100 µm. **(b)** Representative voltage-clamp recording from a VGAT neuron expressing ChR2, showing the characteristic ChR2-mediated excitation by a blue light flash (blue vertical line above the trace) at different holding potentials. Protocols and *I*–*V* plots are as in Fig. 6.
